# Unemployment Insurance

**Published:** 1920-10-30

**Authors:** 


### Unemployment Insurance.

There is a good deal of uncertainty about the way in which Unemployment Insurance will affect nurses. A large proportion of nurses would rejoice to hear that a decision had been reached which excepted them as a body from the provisions of the new Act. But there is another side to the picture. In almost every case of a nurse being reduced to distress in the later years of her life she will be found attributing her troubles to intermittent employment. Unemployment Insurance is not intended for the more prosperous members of any profession. It is meant for that stratum of workers who are on the verge of poverty, who can maintain themselves under normal conditions, but have scanty reserves, and are liable at any moment to be beaten by circumstances. To what a large extent this is true of nurses the administrators of the Tribute Fund for Nurses have reason to know. If they are included in the provisions of the Unemployment Insurance Act they will be entitled to receive 12s. a week (after the first three days of unemployment), though more than fifteen weeks' benefit will not be allowed in any one year.

